# Identification of Heat Shock Protein 60 as a Regulator of Neutral Sphingomyelinase 2 and Its Role in Dopamine Uptake

**DOI:** 10.1371/journal.pone.0067216

**Published:** 2013-06-19

**Authors:** Kyong-Hoon Ahn, Seok-Kyun Kim, Jong-Min Choi, Sung-Yun Jung, Jong-Hoon Won, Moon-Jung Back, Zhicheng Fu, Ji-Min Jang, Hae-Chan Ha, Dae-Kyong Kim

**Affiliations:** Department of Environmental & Health Chemistry, College of Pharmacy, Chung-Ang University, Dongjak-Ku, Seoul, South Korea; Max Delbrueck Center for Molecular Medicine, Germany

## Abstract

Activation of sphingomyelinase (SMase) by extracellular stimuli is the major pathway for cellular production of ceramide, a bioactive lipid mediator acting through sphingomyelin (SM) hydrolysis. Previously, we reported the existence of six forms of neutral pH–optimum and Mg^2+^-dependent SMase (N-SMase) in the membrane fractions of bovine brain. Here, we focus on N-SMase ε from salt-extracted membranes. After extensive purification by 12,780-fold with a yield of 1.3%, this enzyme was eventually characterized as N-SMase2. The major single band of 60-kDa molecular mass in the active fractions of the final purification step was identified as heat shock protein 60 (Hsp60) by matrix-assisted laser desorption/ionization time-of-flight mass spectrometric analysis. Proximity ligation assay and immunoprecipitation study showed that Hsp60 interacted with N-SMase2, prompting us to examine the effect of Hsp60 on N-SMase2 and ceramide production. Interestingly, Hsp60 siRNA treatment significantly increased the protein level of N-SMase2 in N-SMase2-overexpressed HEK293 cells. Furthermore, transfection of Hsp60 siRNA into PC12 cells effectively increased both N-SMase activity and ceramide production and increased dopamine re-uptake with paralleled increase. Taken together, these results show that Hsp60 may serve as a negative regulator in N-SMase2-induced dopamine re-uptake by decreasing the protein level of N-SMase2.

## Introduction

Sphingolipids are known to be important components of signaling pathways because their metabolism gives rise to products that function as second messengers, such as ceramide, sphingosine, and sphingosine-1-phosphate. Of these, ceramide, which is a central metabolite, is recognized as an important lipid messenger that plays key roles in a variety of cellular responses, including cell cycle arrest, apoptosis, senescence, and stress responses [1]. In addition, ceramide is known to play physical roles in membrane microdomain function, membrane vesiculation, fusion/fission, and vesicular trafficking [2]. As a lipid second messenger, ceramide is generated in response to a variety of stimuli, including TNF-α [3], Fas ligand [4], phorbol ester [5], heat stress [6], oxidative stress [7], ionizing radiation [8], and chemotherapeutics [9].

Sphingomyelinase (SMase) breaks down sphingomyelin (SM) to produce ceramide and phosphocholine. Several neutral SMase (N-SMase) activities have been described in mammalian brain [10], [11], [12], [13]. By using a bioinformatics-based approach, 47.5-kDa N-SMase1 [14], 71-kDa N-SMase2 [15], and 97-kDa N-SMase3 [16] have been cloned and characterized.

The brain has been shown to have the highest levels of enzymatic N-SMase activity, suggesting that N-SMase may regulate brain-specific processes [17]. However, the precise roles of these enzymes in the brain and the mechanisms by which they function remain incompletely understood. N-SMase2 is one of the N-SMases found mainly in the brain [15], [18], and it has been implicated in apoptosis, inflammation, and cell growth [19]. Although most N-SMase2 studies have focused on apoptosis and cell cycle arrest, our recent study was the first to suggest a role of N-SMase2 in dopamine (DA) re-uptake [20]; N-SMase2 could be a modulator of DA re-uptake through its ability to regulate intracellular calcium in dopaminergic neurons.

Cumulatively, these findings imply that multiple forms of N-SMase may exist in the brain and exert their roles through a concerted mechanism in which a variety of factors crosstalk. Previously, we reported the identification and partial purification of six forms of N-SMase from the membrane fractions of bovine brain, including four (N-SMase α, β, γ, and δ) from Triton X-100 extracts and two (N-SMase ε and ζ) from salt extracts [13]. Since N-SMase2 is already known to be extracted with Triton X-100, we focused on salt-extracted N-SMase, which may be a novel form in the membrane fractions of bovine brain.

In the present study, N-SMase ε was extensively purified by 12,780-fold with a 1.3% yield from the salt extracts; it was seen as a major single band of 60-kDa molecular mass with paralleled activity in silver stained gel. This 60-kDa protein was identified as heat shock protein 60 (Hsp60) by matrix-assisted laser desorption/ionization time-of-flight (MALDI-TOF) mass spectrometric analysis. However, overexpression of Hsp60 in yeast did not show an increase in N-SMase activity, and the major activity in the active fractions of the final chromatographic step showed similar biochemical properties and antigenic cross-reactivity with N-SMase2. These results suggest that N-SMase ε could be identical to N-SMase2 and that Hsp60 may serve as a regulatory protein bound to N-SMase2. Our data further showed that Hsp60 serves as a negative regulator for N-SMase2–induced DA re-uptake by inducing a decrease in the protein N-SMase2, thereby reducing ceramide generation.

## Materials and Methods

### Materials

We purchased [^3^H] palmitic acid (1.2 Ci/mmol), [*N-methyl*-^14^C] SM (48.5 Ci/mmol), and [^3^H] DA (4 Ci/mmol) from Amersham Pharmacia Biotech UK Ltd. (Buckinghamshire, England). DTT, SDS, and CHAPS were purchased from Sigma (St. Louis, MO). All equipment for isoelectric focusing and SDS-PAGE, the immobilized pH gradient (IPG) buffer (pH 4–7) and IPG strips (pH 4–7) were obtained from Amersham Pharmacia Biotech (Uppsala, Sweden). Silica Gel 60 thin-layer chromatography plates were obtained from Merck (Darmstadt, Germany). The DE52 anion-exchange gel was purchased from Whatman (Maidstone, UK). Butyl-Toyopearl hydrophobic resin, the DEAE-5PW anion-exchange HPLC column, and the phenyl-5PW hydrophobic HPLC column were purchased from Tosho Co. (Tokyo, Japan). The Mono S cation-exchange column and the Superose 12 gel filtration column were purchased form Amersham Biosciences. Unless otherwise stated, all the reagents used in this study were of the highest purity and were purchased from Sigma.

### Purification of N-SMase ε from bovine brain

Similar to the protocol described previously for purification of N-SMase α, β, γ, and δ [13], a salt-extractable form of membrane-bound N-SMase ε was purified as follows. First, to prepare the enzyme source for purification of N-SMase, fresh bovine brain (5 kg) kept at −70°C was homogenized with five volumes (25 l) of homogenizing buffer V (50 mM Tris-HCl, pH 7.5; 1 mM EDTA, 3 mM MgCl_2_, 50 mM KCl, and 10 mM 2-mercaptoethanol) using a Polytron homogenizer (Model PT-MR 6000; Kinematica, Switzerland). The homogenate was centrifuged at 10,000× *g* for 10 min to remove cell debris and nuclei. The resulting supernatants were again centrifuged at 10,000× *g* at 4°C for 1 h. The pellets thus obtained were resuspended with 2.5 l of buffer V, adjusted to 0.5 M (NH_4_)_2_SO_4_, and stirred at 4°C for 1 h, followed by centrifugation at 40,000× *g* at 4°C for 1 h. The resulting supernatants, termed “ammonium sulfate extracts,” were collected and used as the enzyme source for the purification of N-SMase ε. The ammonium sulfate extracts (2.5 l) were applied to a DEAE-cellulose column (bed volume of DE52 gel, 1.0 l) pre-equilibrated with buffer D (25 mM Tris-HCl, pH 7.5; 1 mM EDTA and 10 mM 2-mercaptoethanol). The protein bound to the column was eluted at a flow rate of 20 ml/min, with stepwise application of buffer D containing 0.5 M (NH_4_)_2_SO_4_ and 0.1% Triton X-100. The active fractions were pooled and sonicated six times for 3 s each at 5-s intervals at an output setting of 70% amplitude at 4°C using a cell disruptor (Sonics & Materials Inc., Danbury, CT), followed by centrifugation at 100,000× *g* at 4°C for 1 h. The resulting supernatant was applied to the butyl-Toyopearl column (bed volume, 150 ml) pre-equilibrated with buffer D containing 0.5 M (NH_4_)_2_SO_4_. The protein bound to the column was eluted at a flow rate of 15 ml/min with sequential stepwise elution of buffer D containing 0.2 M (NH_4_)_2_SO_4_, followed by application of distilled water. The active fractions were pooled and applied to the DEAE-5PW HPLC column (21.5 mm ×15 cm; Tosoh Co., Tokyo, Japan) pre-equilibrated with buffer D. The protein bound to the column was eluted at a flow rate of 5 ml/min with a 200-ml linear gradient of buffer D containing 0.5 M (NH_4_)_2_SO_4_ and 0.1% Triton X-100 as the elution buffer. The active fractions were pooled and applied to the Phenyl-5PW HPLC column (21.5 mm ×15 cm; Tosoh Co., Tokyo, Japan) pre-equilibrated with buffer D containing 0.2 M (NH_4_)_2_SO_4_. The protein bound to the column was eluted at a flow rate of 5 ml/min with a 200 ml-gradient elution of distilled water. The active pool was mixed with the same volume of buffer S (25 mM sodium acetate, pH 6.5, and 1 mM EDTA) and applied to the Mono S cationic exchange FPLC column (5.0 cm ×5.0 mm) pre-equilibrated with buffer S. The protein bound to the column was eluted at a flow rate of 1 ml/min with a 20-ml linear gradient of 0.0–1.0 M NaCl followed by a stepwise gradient of buffer S containing 1.0 M NaCl and 0.1% Triton X-100. An aliquot (3 µl) of each fraction (1 ml) was assayed for N-SMase activity.

### Cell culture and transfection

HEK293 and PC12 cells were obtained from the American Type Culture Collection. PC12 cells were maintained in 10% horse serum and 5% FBS in RPMI 1640 (Invitrogen, Carlsbad, CA) at 37°C in 5% CO_2_. For siRNA experiments, cells were seeded into poly-D-lysine-coated plates (2×10^5^ cells/well). After 24 h, cells were transfected with scramble or specific siRNA (20 nM) using Lipofectamine RNAiMAX (Invitrogen) according to the manufacturer's protocol. Cells were allowed to grow for 48 h before experiments. Prevalidated siRNA for Hsp60 and N-SMase2 were purchased from Dharmacon (Lafayette, CO) and Invitrogen, respectively.

HEK293 cells were maintained in 10% FBS in DMEM (Invitrogen) at 37°C in 5% CO_2_. For the siRNA experiments, cells were seeded into six-well plates (2×10^5^ cells/well). After 24 h, the cells were transfected with scramble or siRNA for Hsp60 using Lipofectamine™ RNAiMAX according to the manufacturer's protocol. Cells were allowed to grow for 48 h, and transient transfections using pRC/N-SMase2 (kindly provided by Yusuf Hannun, Medical University of South Carolina, Charleston, SC) were performed using Lipofectamine 2000 reagent (Invitrogen) according to the manufacturer's protocol. The cells were further grown for 12–60 h after transfection before experiments.

### Immunoprecipitation (IP) and chemical crosslinking

Anti-Hsp60 monoclonal antibody (StressGen, Vancouver, Canada) and anti-N-SMase2 rabbit polyclonal or mouse monoclonal antibody (Santa Cruz Biotechnology, Santa Cruz, CA) were mixed with Protein A Sepharose beads (Amersham Biosciences) and incubated overnight at 4°C with constant shaking. The beads were washed six times with 20 mM Tris-HCl (pH 7.5) containing 1 mM EDTA and 5 mg/ml BSA. Protein A Sepharose beads of various amounts were incubated with the active pool of purified N-SMase or proteins from cell lysate, for 4 h at 4°C with constant shaking. The beads were then pelleted and washed five times with 20 mM Tris-HCl (pH 7.5) containing 1 mM EDTA. Equal aliquots from the supernatants and the washed immunoprecipitates were assayed for N-SMase activity. The supernatant and immunoprecipitates were separated in 10% SDS-PAGE gel, transferred to nitrocellulose membrane, and probed with anti-Hsp60 or N-SMase2 antibody.

Crosslinking was performed according to the standard method described in the manufacturer's protocol. Briefly, harvested cells were resuspended in PBS (2 ml) and combined with 20 µl of the crosslinker dithiobis (succinimidylpropionate) (DSP; Pierce, Rockford, IL) stock solution in dimethyl sulfoxide to a final concentration of 2 mM. After incubation of the reaction mixture on ice for 1 h, the cells were rinsed with PBS and solubilized on ice for 30 min in buffer (25 mM Tris-HCl, pH 7.5; 2 mM EDTA, 0.1% Triton X-100, 137 mM NaCl, protease inhibitor cocktail, and phosphatase inhibitor cocktail). The extracts were centrifuged for 15 min at 10,000× *g*, and the supernatants were pre-cleared with Protein A Sepharose for 1 h. Pre-cleared supernatants were kept on ice for bicinchoninic acid protein assay and IP.

### In Vitro SMase assay

Sphingomyelinase activity was determined as described previously [20]. Briefly, the standard incubation system (100 µl) for the assay of N-SMase activity contained 10 mM MgCl_2_, 5.0 µM [N-*methyl*-^14^C] SM (labeled with ^14^C on the choline moiety), 2 mM sodium deoxycholate or 0.02% Triton X-100, and 100 mM Tris-HCl (pH 7.5). Reactions were carried out at 37°C for 10 min and stopped by adding 320 µl of chloroform/methanol (1∶1 volume) and 30 µl of 2 M HCl. After vortexing and centrifugation, 200 µl of the clear aqueous phase was added to 2.5 ml scintillation solution (Insta gel-XF; Packard Instrument Co., Meriden, CT, USA) and then radioactivity was counted using a Packard Tri-carb liquid-scintillation counter. In order to characterize the calcium dependence, an aliquot of the active fractions was applied to a PD-10 desalting column (Pharmacia LKB, Uppsala, Sweden), pre-equilibrated with 25 mM Tris−HCl (pH 7.5) containing 0.1% Triton X-100 and 10 mM 2-mercaptoethanol. The N-SMase activity of the desalted fractions was assayed in the presence of 0–1 mM MgCl_2_ at the Ca^2+^ concentrations indicated. The absolute concentrations of free Ca^2+^ were calculated using an equation based on the stability constant of the EGTA/CaCl_2_ system. Enzyme characteristics in the presence of PS and GW4869 were evaluated as described previously [21].

The N-SMase activity of the immunoprecipitates was assayed in the absence or presence of the indicated concentrations of cations or GW4869 with constant shaking.

### Preparation of rat brain synaptosomes and mitochondria

Adult Sprague-Dawley rat, which were used for obtaining tissues, were purchased from Orient Bio (Orient Bio, South Korea). All animal experiments were performed in accordance with the National Research Council's Guidelines for the Care and Use of Laboratory Animals and with the Guidelines for Animal Experiments of Chung-Ang University, and were approved by the University Committee for animal experiments. Rat Brains were rapidly removed and brain synaptosomes were prepared as described previously [22]. Isolation of cortical non-synaptosomal brain mitochondria was achieved by using a discontinuous Percoll gradient as described previously [23]. All steps were carried out under ice-cold conditions. Protein quantification of synaptosomal and mitochondrial suspensions was performed by the Bradford method using BSA as a standard.

### Proteomics analysis

Two-dimensional gel electrophoresis was performed according to O′Farrell [24] using the IPG-phor system (Amersham Pharmacia Biotech) according to the manufacturer's instructions. The separated proteins were stained by using a PlusOne silver staining kit (Pharmacia Biotech Inc., Piscataway, NJ). An aliquot of the total digest was used for peptide mass fingerprinting. Masses were measured with a Bruker Reflex IV mass spectrometer (Bruker Daltonik, Bremen, Germany) equipped with a 337-nm nitrogen laser and operated in positive ion reflector mode. MALDI-TOF analysis was performed using α-cyano-4-hydroxycinnamic acid as the matrix. Spectra were acquired over 750–3000 m/z and calibrated externally using the peptide calibration standard (Bruker Daltonik). Proteins were identified by peptide mass fingerprinting with the search engine programs ProFound (Laboratory of Mass Spectrometry and Gaseous Ion Chemistry, Rockefeller University, New York, NY) and Mascot (Matrix Science, Inc., Boston, MA).

### Real-time RT-PCR for N-SMase2

Total cellular RNA was isolated from PC12 cells using the TRI-Reagent protocol (Molecular Research Center Inc., Cincinnati, OH). Real-time PCR was performed using an ABI 7900 HT Fast Real-Time PCR system (Applied Biosystems, Foster City, CA). The primers were designed for rat N-SMase2 (right, 5′-TGAAAACATT-ATTGAGCCTTGC-3′, and left, 5′-CTTTGCCACAGCCAATGTC-3′) and β-actin (right, 5′-TCAGGCAGCTCATAGCTCTTC-3′, and left, 5′-GCCCTAGACTTCGAG-CAAGA-3′) as previously described [25].

### Assay for [^3^H] dopamine release and uptake in PC12 cells

The levels of dopamine release and uptake were determined as described previously [20], [26].

### Determination of intracellular ceramide levels

The level of ceramide was determined as described previously [26]. Briefly, PC12 cells (2×10^5^) growing in six-well plates were labeled with 1 µM [^3^H] palmitic acid (1 µCi/ml; Amersham) and, after 24 h, the cells were washed twice with PBS and collected in 400 µl of an acidified methanol solution (CH_3_OH∶0.5-N HCl  = 1∶1) and 200 µl chloroform, vortexed vigorously for 20 min, and centrifuged at 15,000× *g* for 30 min at 4°C. The chloroform phase was collected and dried under a nitrogen stream. The lipids were dissolved in 20 µl of a chloroform-methanol solution (1∶1; v/v) and separated with non-radioactive ceramide using TLC with a solvent system containing chloroform:methanol:acetic acid:water (21.25∶1.125∶1.25∶0.125, respectively; v/v). Ceramide spots were visualized using iodine vapor and then scraped. Radioactivity was measured using a β-scintillation counter (Tri-carb 1600 TR; Packard Instrument Co., Meriden, CT).

### LC-MS/MS

Measurement of ceramide subspecies was performed by LC/MS/MS as described previously [27]. The HPLC system consisted of an Agilent 1100 series binary pump and a column oven (Agilent technologies Inc., Santa Clara, SC, USA), combined with a CTC PAL autosampler (CTC Analytics, Zwingen, Switzerland). The analytical column was an X Terra MS C18 (2.0×50 mm, 3 µm; Waters Co.) and was kept at 45±0.5°C during the analysis. The mobile phase consisted of 0.1% formic acid and methanol buffered with 2 mM ammonium formate/tetrahydrofuran (7/3, v/v; hereafter referred to as A) and 5 mM ammonium formate (pH 4.0)/methanol (9/1, v/v; hereafter referred to as B). For HPLC separation, a gradient program was used at a flow rate of 200 µl/min. The initial buffer composition was 80% mobile A and 20% mobile B, which was then linearly changed to 100% mobile A after 5 min and maintained for 4 min, and was then immediately reverted to the initial condition and maintained for 11 min. The run time of each experiment was 20 min. The HPLC system was coupled online to an SCIEX API 3000 triple-quadruple mass spectrometer (Applied Biosystems, Concord, Canada) equipped with a Turbo Ion Spray source. The mass spectrometer operated with unit resolution for both Q1 and Q3. Multiple reactions monitoring was employed using nitrogen as the collision gas with a dwell time of 150 ms for each transition. The optimal mass spectrometry values were obtained by manual tuning or automatic tuning, with each standard injected by a syringe pump. Data acquisition was confirmed using Analyst 1.4.1 software. The calibration curves for the sphingolipid standards were generated by plotting the peak area ratio (analyte/IS) versus the concentrations in the calibration standard samples by least-square linear regression.

### Western blot analysis

Proteins were separated by SDS-PAGE (10% or 12% gels) and then electrophoretically transferred to nitrocellulose membranes (Schleicher & Schuell, Keene, NH). The membrane was then blocked for 1 h at room temperature in 5% non-fat milk in TBS (20 mM Tris-HCl, pH 7.4; 137 mM NaCl and 2.7 mM KCl). The blocked nitrocellulose membranes were incubated with antibodies against N-SMase2 (1∶2000; Santa Cruz Biotechnology), GAPDH (1∶2500; Bethyl Laboratories, Montgomery, TX), and Hsp60 (1∶2500; StressGen, Enzo Life Sciences, Plymouth Meeting, PA) at room temperature with constant shaking. The sites of antibody binding were detected using an alkaline phosphatase-conjugated goat anti-rabbit IgG antibody (Santa Cruz Biotechnology) and a chromogenic substrate (1-StepTM NBT/BCIP; Pierce).

### In situ proximity ligation assay

The co-localization of N-SMasae2 and HSP60 in HEK293 cells which were overexpressed with N-SMase2 was determined with proximity ligation assay (PLA assay). Briefly, HEK293 cells (2.5×10^5^) were transfected with N-SMase2 as described above. Then cells were grown on eight-well chamber slides overnight. Cells were fixed with formaldehyde prepared in PBS (4%, 20 min). Cells were perrmeabilized with 0.5% Triton X-100 prepared in DPBS (20 min, room temperature), and blocked for 30 min, 37°C in Duolink blocking solution (Duolink In Situ kit, Olink Bioscience). Cells were incubated overnight at 4°C with pairs of primary antibodies in Duolink antibody diluent solution; i.e. mouse anti-N-SMase2 (Santa Cruz Biotechnology, Inc.) and goat anti-Hsp 60 (Santa Cruz Biotechnology, Inc.). PLA probes were diluted in 0.1% Triton X-100/1% FCS/PBS and incubated in preheated humidity chamber for 1 h at 37°C, followed by hybridization, ligation, amplification and detection according to the manufacturer's protocol. The concatemeric amplification products extending from the oligonucleotide arm of the PLA probes were then detected using a confocal scanning microscope (LSM510, Zeiss).

### Statistical analysis

Data are presented as mean values ± standard deviation (S.D.) of the indicated number of experiments. One-way ANOVA or Student's *t*-test was used for statistical analysis. A *p* value <0.05 was considered statistically significant.

## Results

### Purification of (NH_4_)_2_SO_4_-extractable, membrane-associated, Mg^2+^-dependent neutral SMase

Based on our previous identification of multiple forms of N-SMase from bovine brain [13], N-SMase can be classified into two groups according to the agent solubilizing the activity in the membrane fractions: (1) a Triton X-100-extractable N-SMase which further consists of four forms of N-SMase (α, β, γ, and δ) and (2) a salt-extractable N-SMase which further consists of two forms of N-SMase (namely N-SMase ε and ζ). In the present study, to purify and characterize N-SMase ε, we obtained ammonium sulfate extract from a 40,000× *g* pellet of bovine brain homogenate. This process resulted in a 4-fold increase in the specific activity of N-SMase over that of the 40,000× *g* pellets as well as removal of the four forms of Triton X-100-extractable N-SMase.

**Figure 1:**
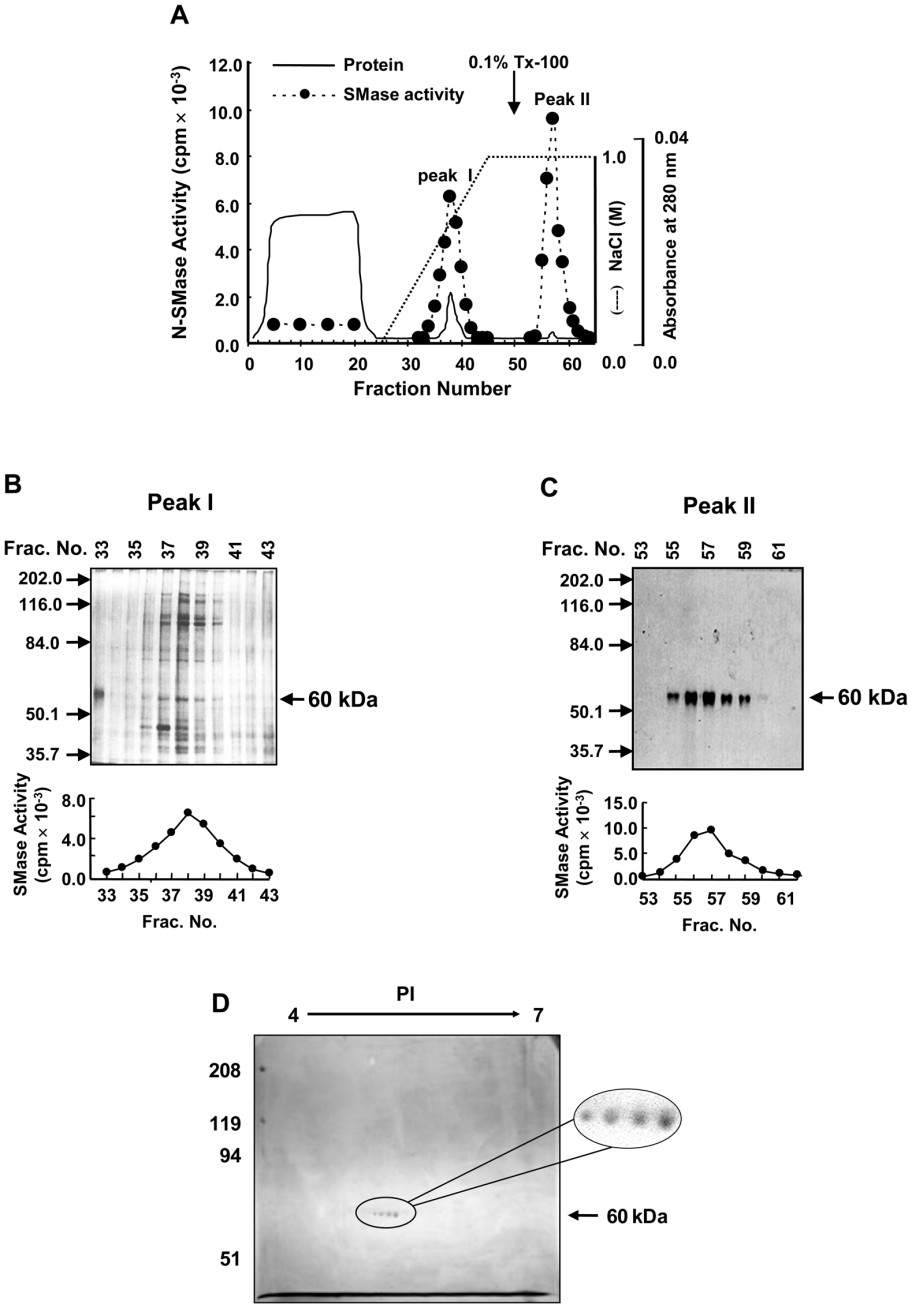
Purification of (NH_4_)_2_SO_4_-extractable, membrane-associated, Mg^2+^-dependent neutral SMase from bovine brain. A membrane-associated neutral form of N-SMase was extracted with 0.2 M (NH_4_)_2_SO_4_ from 10,000× *g* pellets of bovine brain homogenates and purified by sequential chromatography steps. (*A*) Mono S cationic exchange FPLC and peak II active fractions were applied to Superose 12 gel filtration chromatography. (*B*) The active fraction purified from Superose 12 gel filtration chromatography was analyzed using two-dimensional gel electrophoresis. The results from this purification scheme are representative of 10 independent experiments.

Next, the N-SMase in the ammonium sulfate extract was purified by sequential use of anion-exchange (DEAE-cellulose), hydrophobic interaction columns (butyl-Toyopearl), anion-exchange HPLC (DEAE-5PW), hydrophobic HPLC (Phenyl-5PW), and cation-exchange FPLC (Mono S). In the final chromatographic process, the sample was bound to the column and eluted as a single 220- to 380-mM peak, which we termed the “salt-eluted peak I.” However, additional N-SMase activity with a tiny protein peak was isocratically eluted with buffer S containing 0.1% Triton X-100 and 1.0 M NaCl, which we termed “Triton X-100-eluted peak II.” This additional peak II showed a specific activity of >1,150 nmol/min/mg, which was higher than that of peak I (275 nmol/min/mg) with a 1.4-fold greater total activity than that of peak I (Figure 1A). These sequential chromatographies for peak I and peak II resulted in 2,000- and 12,780-fold purification, respectively, from the 40,000× *g* pellets of bovine brain homogenate.

To assess their purity, purified peak I and peak II active fractions were analyzed for protein distribution by SDS-PAGE, followed by silver staining. Peak I active fractions migrated as several bands, among which, only the 60-kDa band correlated with N-SMase activity (Figure 1B), whereas peak II active fractions revealed one major protein species with molecular masses of 60-kDa (Figure 1C); these were designated as N-SMase ε. In some preparations also minor protein bands corresponding to molecular masses of 57, 71 and 82-kDa, respectively, were also occasionally observed. The most active fraction, which was purified by an additional purification step (i.e., the Superose 12 gel filtration columns), was separated into four distinguishable spots in a two-dimensional electrophoresis gel (Figure 1D). All four spots were excised from the gel and analyzed by MALDI-TOF. A database search using the obtained monoisotopic peptide masses allowed us to identify these spots as Hsp60. In order to establish that Hsp60 is a structural gene for N-SMase, we expressed Hsp60 into the JK9-3d yeast (*MATα/α trp1 leu2-3 his4 ura3 ade2rme1*) strain with deletion of *ISC1*, which encodes for an endogenous inositol phosphosphingolipid phospholipase C [28]. Overexpression of Hsp60 in the yeast was not higher than that in the vector-transfected cells (Figure S1).

### Characterization and identification of purified N-SMase ε as N-SMase2

**Figure 2:**
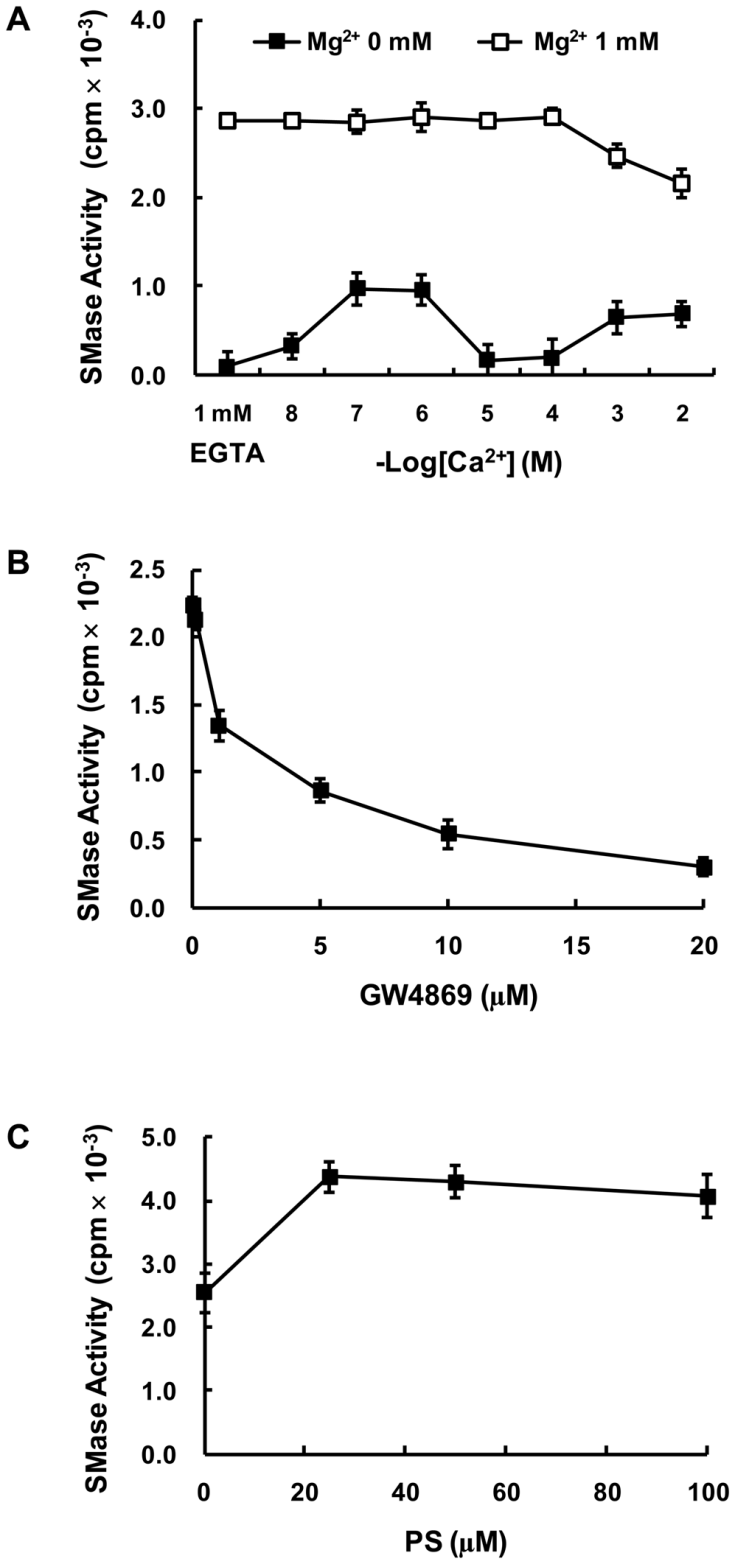
Effects of cationic ions, GW4869 and PS on purified N-SMase. Purified N-SMase activity was determined in the presence of different concentrations of Ca^2+^, with or without 1 mM Mg^2+^ (*A*). Purified N-SMase was pre-incubated with the indicated concentrations of GW4869 (*B*) or PS (*C*) for 10 min at 37°C, and then activity was measured with the standard assay system. These results represent means ± S.D. from a single experiment performed in triplicate. Similar results were obtained in three independent experiments.

**Figure 3:**
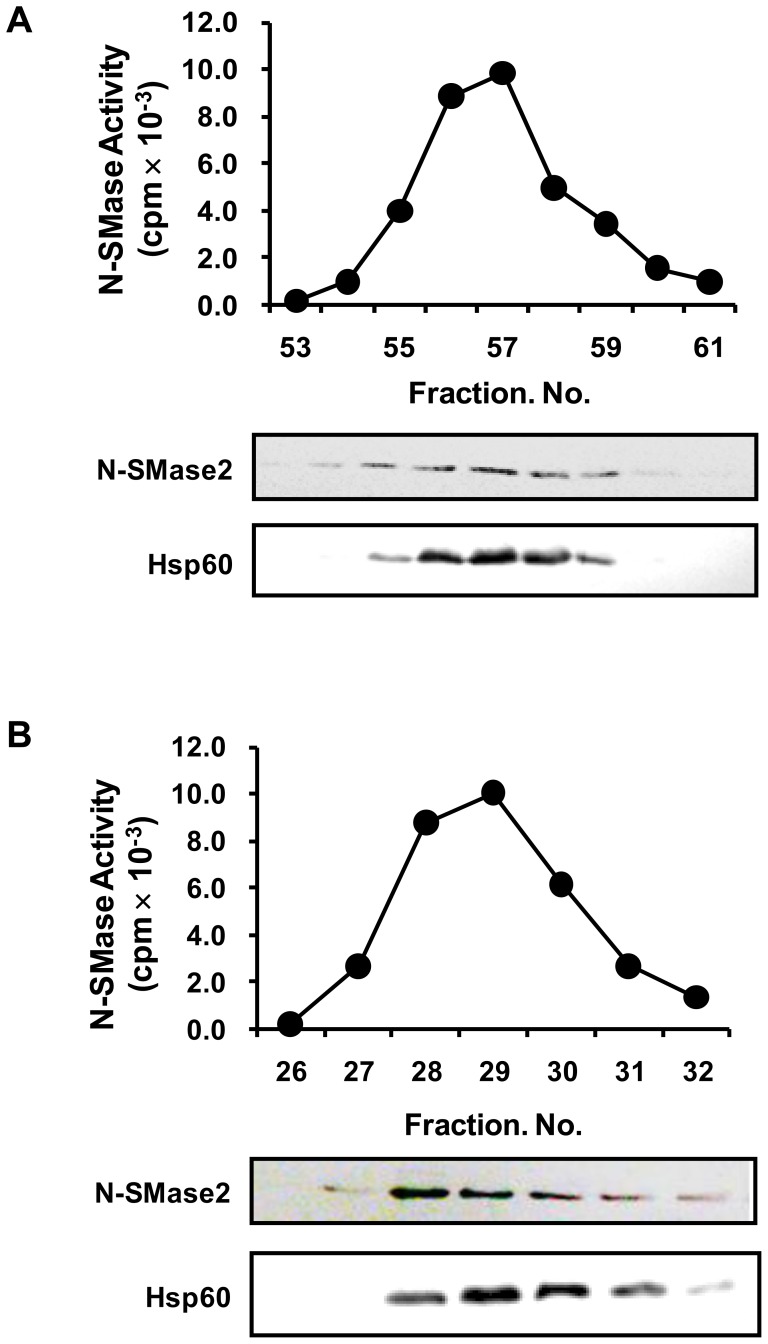
Western blotting indicates that high N-SMase2 levels coincide with N-SMase activity. Equal volumes of active N-SMase fractions from the Mono S FPLC (*A*) and Superose 12 gel filtration (*B*) columns were assayed for N-SMase activity, and the presence of N-SMase2 was evaluated by western blot analysis. The results are from a single experiment, which was representative of five different experiments.

The active fractions obtained from the Mono S step (peak II) were used for characterization of the N-SMase activity. The activity of purified N-SMase ε was dependent upon Mg^2+^ and Mn^2+^, whereas Cu^2+^, Zn^2+^ and Ni^2+^ were unable to stimulate activity (data not shown). N-SMase ε was not activated by Ca^2+^ cations in the presence of 1 mM Mg^2+^, but could be activated by 10^−7^ or 10^−6^ M Ca^2+^, in the absence of Mg^2+^ (Figure 2A). Purified N-SMase ε was inhibited by GW4869 and activated by phosphatidylserine (PS) (Figure 2B and C). Because the Ca^2+^ and PS effects were similar to that of N-SMase2 as reported in our previous study [18], the active fractions (peak II) from the Mono S (Figure 3A) and Superose-12 columns (Figure 3B) were separated in a 10% SDS-PAGE gel and probed with the anti-N-SMase2 or anti-Hsp60 antibody. As shown in Figure 3, the enzyme activity and N-SMase2 western blot band showed a parallel relation, suggesting that purified N-SMase ε could be identified as N-SMase2. Based on these observations, we hypothesized that Hsp60 can interact with N-SMase2 in a purified sample from bovine brain.

### Immunoprecipitation of N-SMase 2 with anti-Hsp60 antibody

**Figure 4:**
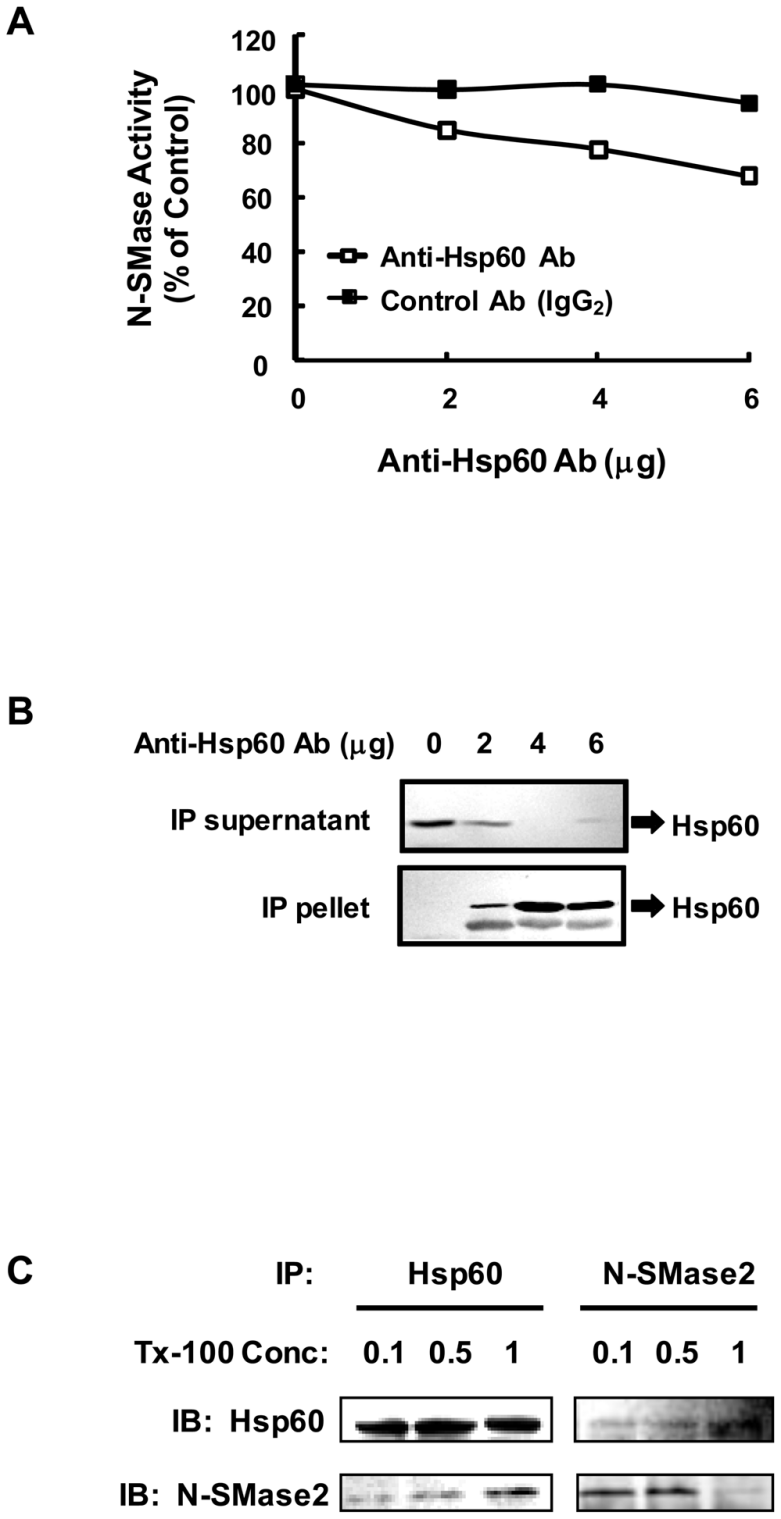
Immunoprecipitation of N-SMase with antibody against Hsp60. (*A*) The active N-SMase fraction from Mono S was used for IP. The active fraction was mixed with the specific antibody for Hsp60. Aliquots from the immunoprecipitated supernatants were assayed for N-SMase activity. (*B*) The supernatant and immunoprecipitates were separated in 10% SDS-PAGE gel, transferred to nitrocellulose membrane, and probed with anti-Hsp60 antibody. (*C*) The active fraction was mixed with indicated concentrations of Triton X-100, and the respective specific antibodies for Hsp60 and N-SMase2 were added. The immunoprecipitated pellets were washed and aliquots of the immunoprecipitates were separated in a 10% SDS-PAGE gel, transferred onto a nitrocellulose membrane, and probed with anti-Hsp60 and anti-N-SMase2 antibodies.

In order to investigate the possibility of an interaction between Hsp60 and N-SMase 2, co-IP studies were performed on purified fractions from bovine brain. Whereas incubation of the Mono S active fraction with increasing amounts of mouse Ig G_2_ (negative control) did not result in any loss of N-SMase activity, antibody against Hsp60 immunoprecipitated N-SMase activity in a dose-dependent manner (Fig 4A, B). As shown in Figure 4A, 4 µg of anti-Hsp60 Ab precipitated 20% of the N-SMase activity. Some 10% of total N-SMase activity could be recovered in the immunoprecipitates after resuspension in IP buffer (data not shown).

In the purification procedure, highly purified N-SMase ε appeared to migrate as a complex on a gel filtration column (data not shown). Moreover, the enzyme displayed highly hydrophobic properties and dependence on detergent. For that reason, we investigated the effect of Triton X-100 on N-SMase activity of the immunoprecipitates. The purified fraction was added to an IP buffer containing the indicated concentration of Triton X-100 and then incubated with anti-Hsp60 or anti-N-SMase2 antibody. When the immunoprecipitates were subjected to the N-SMase assay, a Triton X-100 concentration–dependent decrease in the activity of N-SMase was revealed (data not shown). On the other hand, western blotting of the immunoprecipitates obtained following IP with Triton X-100 revealed no differences (Figure 4C).

### Characterization of the N-SMase in immunoprecipitates purified from bovine brain using Hsp60 antibody

**Figure 5:**
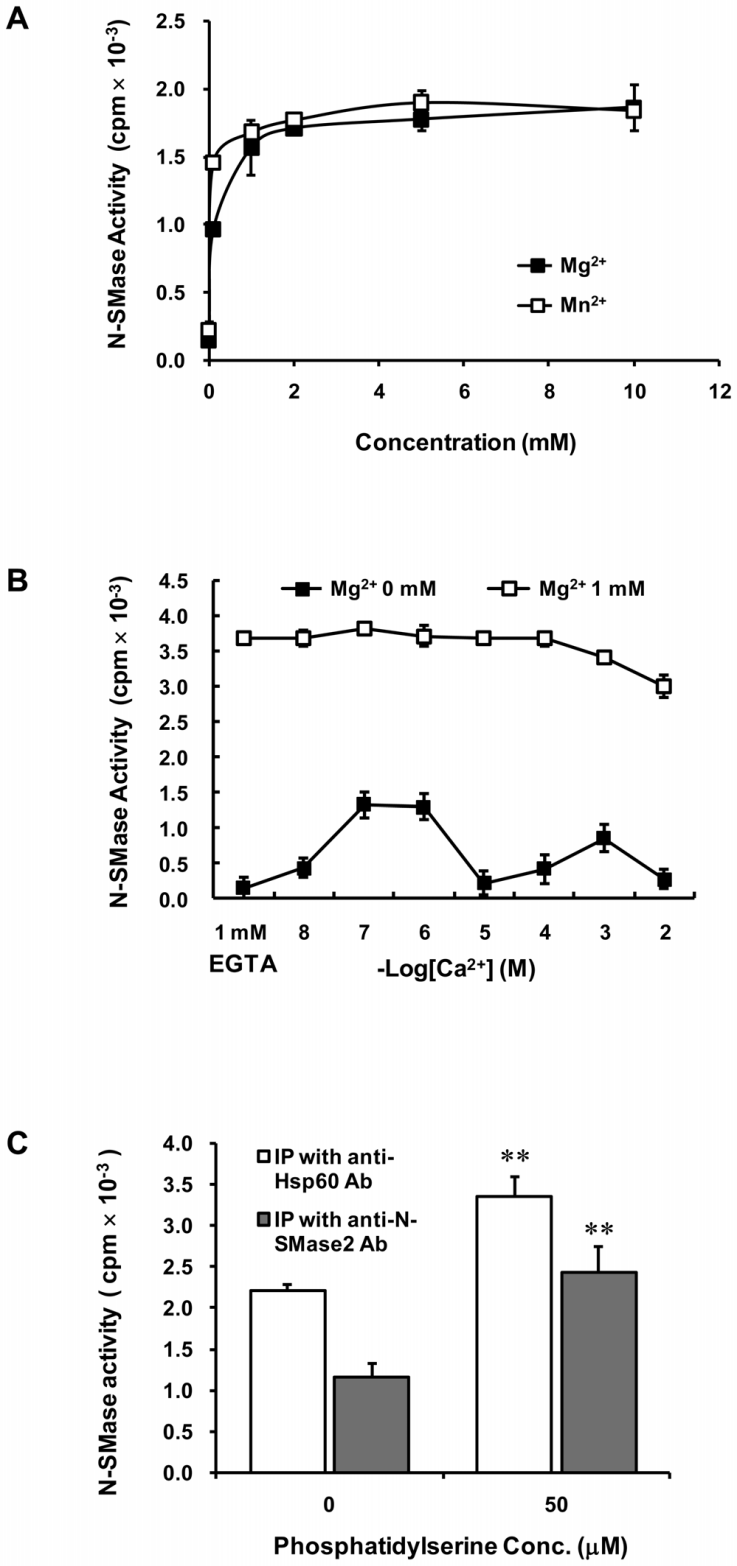
Effects of cations and PS on N-SMase activity of anti-Hsp60 antibody immunoprecipitates. The active fraction from Superose 12 gel filtration column was mixed with the specific antibody for Hsp60. The immunoprecipitated pellets were washed and aliquots of the immunoprecipitates were assayed for N-SMase activity. N-SMase activity was determined in the presence of different concentrations of Mg^2+^ and Mn^2+^ (*A*). N-SMase activity was determined in the presence of different concentrations of Ca^2+^ with or without 1 mM Mg^2+^ (*B*). The immunoprecipitates with the specific antibody for Hsp60 and N-SMase2 were pre-incubated with 50 µM PS for 10 min at 37°C, and then activity was measured with the standard assay system (*C*). These results represent mean ± S.D. from a single experiment performed in triplicate. Similar results were obtained in five independent experiments.

In order to confirm whether Hsp60 interact with N-SMase2, the pellets immunoprecipitated with anti-Hsp60 antibody were used for characterization of N-SMase. The N-SMase activity of the immunoprecipitates was dependent upon Mg^2+^ and Mn^2+^ (Figure 5A), whereas Cu^2+^, Zn^2+^ and Ni^2+^ were unable to stimulate activity (data not shown). The N-SMase activity of the immunoprecipitates was not activated by Ca^2+^ cation in the presence of 1 mM Mg^2+^, but it could be activated by 10^−6^ and 10^−7^ M Ca^2+^ in the absence of Mg^2+^ (Figure 5B). Interestingly, the N-SMase activity in the immunoprecipitates was activated by PS. Moreover, the IP study using N-SMase2 antibody revealed that the N-SMase activity of the immunoprecipitates was similar to the activity of immunoprecipitates by anti-Hsp60 antibody (Figure 5C). These results strongly suggest Hsp60 can interact with N-SMase2.

### Hsp60 interacts with N-SMase2 on a cellular level

**Figure 6:**
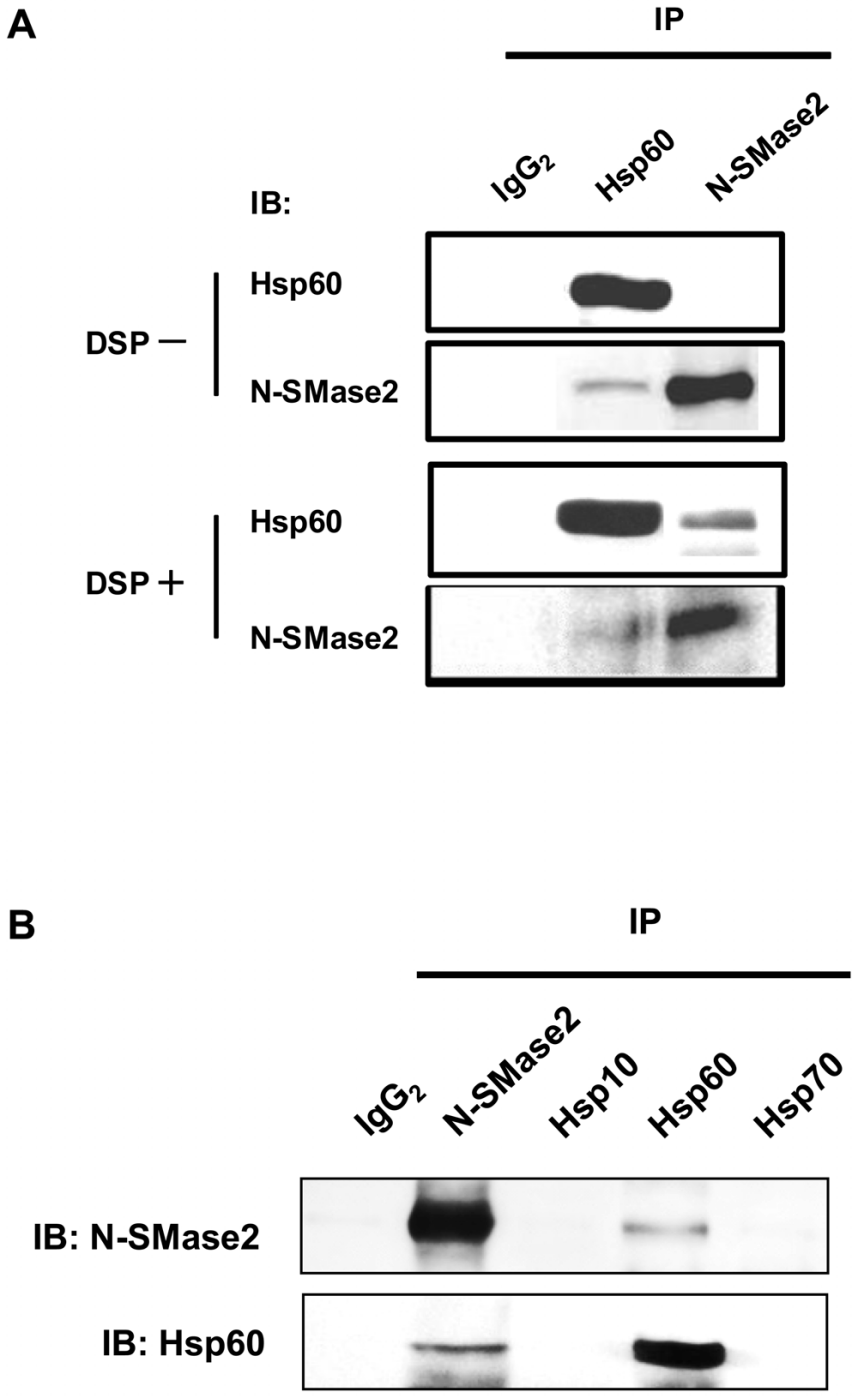
Co-immunoprecipitation of Hsp60 and N-SMase2 in N-SMase2–transfected HEK293 cells. The cells were seeded into six-well dishes, and 24 h later, they were transfected with N-SMase2 plasmids (2 µg/well). After 48 h, harvested cells were resuspended in PBS with or without DSP. Hsp60 and N-SMase2 were immunoprecipitated from cell lysates, as described under “Experimental Procedures.” Aliquots of the immunoprecipitates were analyzed for Hsp60 and N-SMase2 content by SDS-PAGE and immunoblotting (*A*). Also N-SMase2-transfected HEK cells were harvested and treated with DSP. N-SMase, Hsp10, Hsp60 and Hsp70 were immunoprecipitated from the cell lysates as described above and the immunoprecipitates were analyzed for N-SMase2 and Hsp60 by immunoblotting (*B*).

To further validate the interaction between Hsp60 and N-SMase2, HEK293 cells were transfected with N-SMase2, and the cell lysates were immunoprecipitated with antibodies against Hsp60 and N-SMase2. Both antibodies were able to immunoprecipitate the N-SMase activity, whereas mouse Ig G_2_ antibody (control antibody) was unable to immunoprecipitate the N-SMase activity (data not shown). In order to confirm interaction between Hsp60 and N-SMase2, the immunoprecipitate was separated with SDS-PAGE and immunoblotted with each antibody. Although antibodies against Hsp60 and N-SMase2 precipitated a notable amount of N-SMase activity, N-SMase2 antibody did not precipitate Hsp60. Therefore, we used a chemical crosslinker, DSP, for stabilizing intermolecular associations between proteins in HEK293 cells. HEK293 cells were transfected with N-SMase2 and treated with DSP, and then the presence of Hsp60 and N-SMase2 in the immunoprecipitates was confirmed by immunoblotting. In the absence of DSP, N-SMase2 antibody precipitated only N-SMase2, whereas in the presence of DSP, N-SMase2 antibody precipitated a significant amount of Hsp60 (Figure 6A). Furthermore, this interaction was specific to Hsp60 since the antibodies to Hsp10 and Hsp70 did not co-precipitate N-SMase2 (Figure 6B). We also determined this interaction on endogenous level in PC-12 cells. Knocking-down of endogenous N-SMase2 significantly reduced the activity of immunoprecipitate in PC-12 cells (Figure S2A). However, we did not detect the interaction between Hsp60 and N-SMase2 on immunoblotting (Figure S2B). The reason why anti-N-SMase2 antibody could not detect N-SMase2 may be caused by the low levels of N-SMase2 in the PC-12 cells.

### Subcellular localization of Hsp60 and N-SMase2 in rat brain

**Figure 7:**
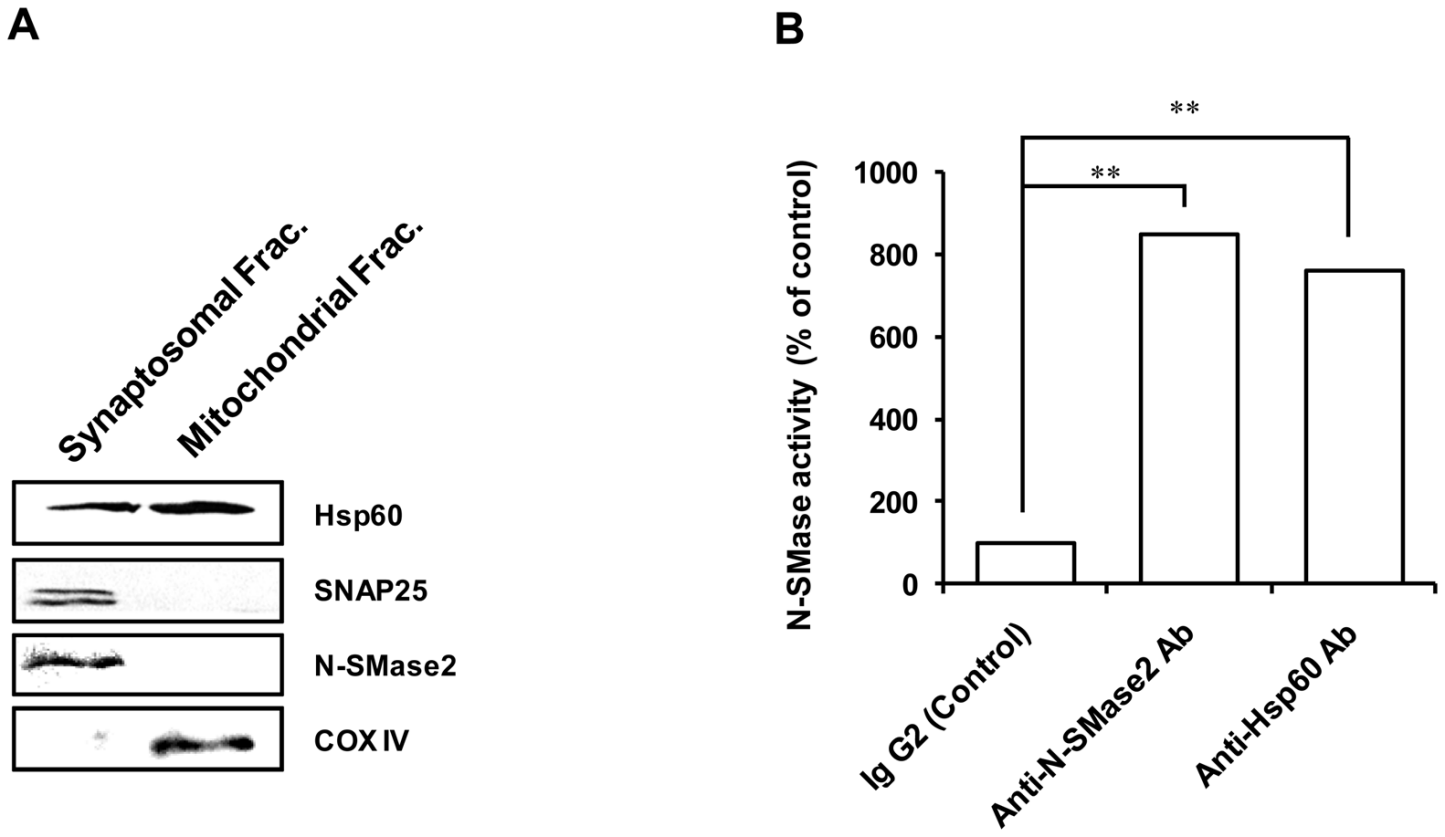
Isolation of synaptosome and mitochondria from rat brain and immunoprecipitation of synaptosome. Isolation of cortical non-synaptosomal brain mitochondria or synaptosome was achieved by using a discontinuous Percoll gradient. Immunoblot (*A*) and IP (*B*) were performed as described under “Experimental Procedures.” The results are representative of three independent experiments that had similar results.

Hsp60 is a common mitochondrial protein with high levels of expression in normal cells. However, Hsp60 is also detectable in extramitochondrial sites, including the endoplasmic reticulum [29], cell surface [30], unidentified vesicles, cytoplasmic granules [31], and lipid rafts [32]. Furthermore, constitutively expressed Hsp90, Hsc70, and Hsp60 were found to be associated with synaptosomes isolated from the brains of unstressed rats [32]. We next conducted studies to establish the subcellular localization of Hsp60 and N-SMase2. Rat brain synaptosomes and mitochondria were prepared and immunoblotted. The purity of the fractions isolated was tested using some marker proteins, i.e., SNAP25 for the synaptosomes and Hsp60 and COX IV for the mitochondrial fraction. As shown in Figure 7A, SNAP25 and N-SMase2 were not detected in mitochondrial fraction, whereas Hsp60 was detected in both fractions.

In order to investigate the interaction of Hsp60 and N-SMase2 on the synaptosome, we performed IP using anti-Hsp60 and anti-N-SMase2 antibodies. As shown in Figure 7B, immunoprecipitates using anti-Hsp60 and N-SMase2 antibodies exhibited a great amount of N-SMase activity compared to control antibody. To confirm that characteristics of immunoprecipitates against anti-Hsp60 antibody were identical to those of N-SMase2, immunoprecipitates were used for characterization of N-SMase as described in Experimental Procedures. In the synaptosome fraction, the N-SMase activity of the immunoprecipitates was dependent upon Mg^2+^ and Mn^2+^. Furthermore, these enzyme activities were activated by Ca^2+^ and PS (data not shown). These results suggest that interaction of N-SMase2 and Hsp60 may regulate neuronal activity in the synaptosome.

### Co-localization of N-SMase2 and Hsp60 in PC-12 cells

**Figure 8:**
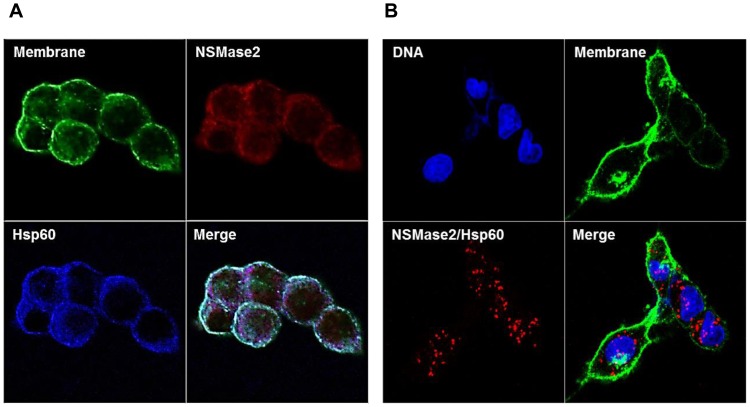
Localization of N-SMase2, Hsp60 and N-SMase2/Hsp60 complex. HEK-293 cells were seeded and transfected with N-SMase2 in 35-mm confocal dishes. After 24 h, (*A*) Cells were fixed and stained with wheat germ agglutinin (green), anti-N-SMase2 (red) and anti-HSP60 (blue) as described under ‘Materials and Methods’. (*B*) On the other hand, the cells were stained with wheat germ agglutinin (green). To visualize a complex of Hsp60 and N-SMase2 as heterodimers we performed an In situ proximity ligation assay using proximity probes against Hsp60 and N-SMase2. The cells were stained with DAPI (blue) to visualize the nucleus.

To confirm the interaction between Hsp60 and N-SMase2, we performed immunocytochemistry studies. First, we confirmed that the N-SMase2 and Hsp60 were detected all around the cell although N-SMase2 was concentrated in plasma membrane more (Figure 8A). Although it seems like to co-localize, it needed to determine more clearly. So, we performed proximity ligation assay to demonstrate the interaction of Hsp60 with N-SMase2 and co-localization. As shown in Figure 8B, the interaction between Hsp60 and N-SMase2 was visualized with red fluorescence spots. No red fluorescence was detected in negative controls stained without anti-N-SMase2 or anti-Hsp60 (Figure S3). Interestingly N-SMase2/Hsp60 complex was located intracellular region (Fig 8B). We expect that Golgi or ER-located Hsp60 would be associated with nearby N-SMase2 and regulate its stability.

### Effect of Hsp60 siRNA transfection on N-SMase activity and ceramide production in PC12 cells

N-SMase activity is approximately 5–6 fold higher in PC12 cells as compared to HEK293 and SH-SY5Y cells, and N-SMase2 staining intensity correlates well with endogenous N-SMase activity [15]. To investigate whether N-SMase2 is responsible for N-SMase activity in PC12 cells and to validate the interaction between Hsp60 and N-SMase2, it was first necessary to confirm that N-SMase2 is detectable in PC12 cells. Accordingly, total cell lysate was immunoblotted for N-SMase2, and IP analysis was performed. We then detected the interaction between Hsp60 and endogenous N-SMase2 in PC12 cells (Figure S2).

**Figure 9:**
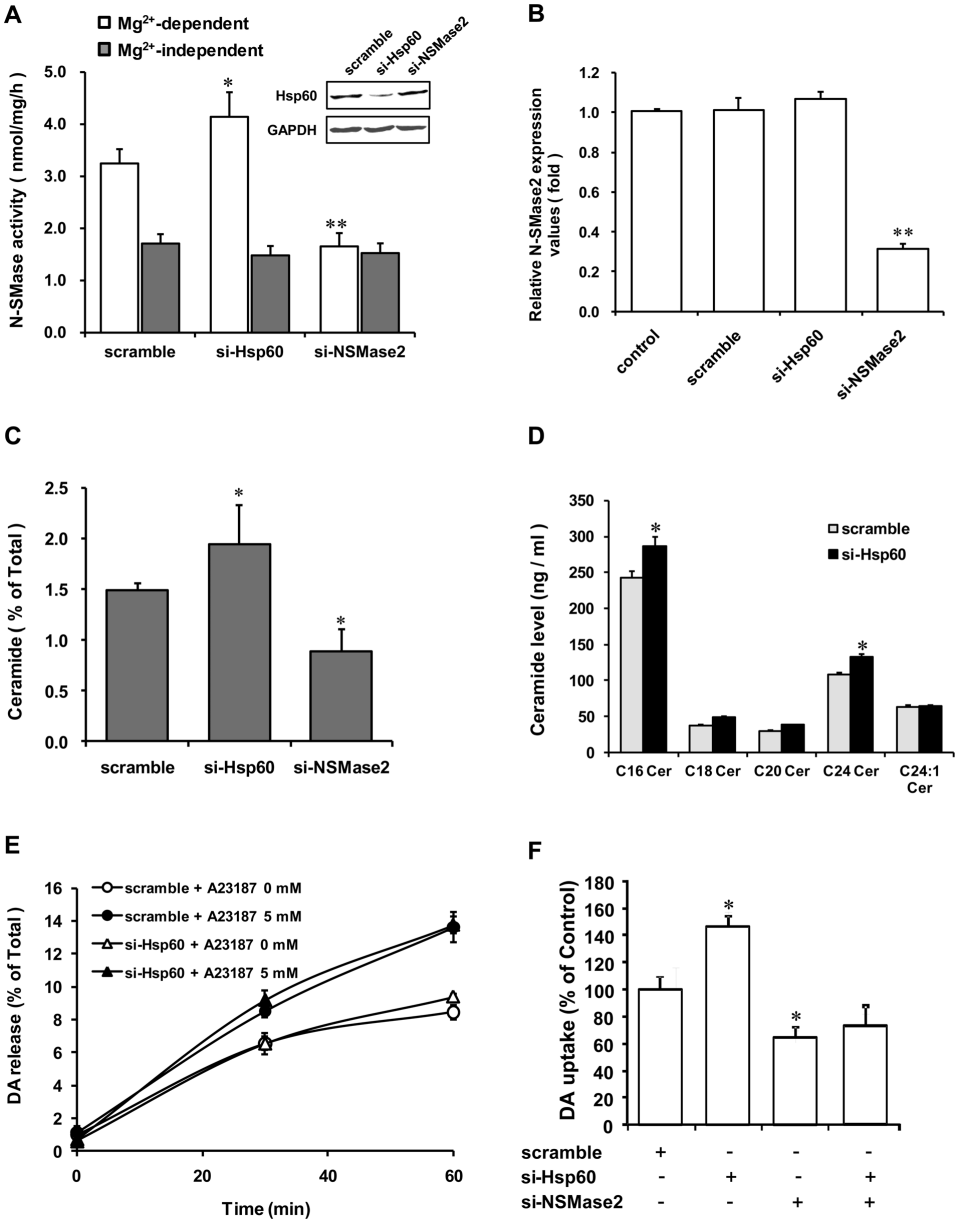
Effect of Hsp60 siRNA on N-SMase activity, ceramide production, and dopamine re-uptake in PC12 cells. PC12 cells were seeded into six-well dishes and transfected with the negative control (scramble) or Hsp60 siRNA (20 nM). After 48 h, the cells lysed, and N-SMase activity was determined. The results represent the mean ± S.D. of seven independent experiments (*A*). After 48 h transfection, total RNA was isolated and purified from the PC12 cells. Real-time PCR was performed using primers specific for N-SMase2 and β-actin (the values are normalized to β-actin) (*B*). After 24 h transfection, the medium was changed, and cells were labeled with [^3^H] palmitic acid. Total lipids were extracted from the PC12 cells and separated with radioactive ceramide using thin-layer chromatography. The ceramide levels in the cells were measured as described under “Experimental Procedures.” (*C*). Scramble, N-SMase2, and Hsp60 siRNA were transfected into PC12 cells, and 48 h later, fresh medium was added with [^3^H] DA. The medium was removed, and the cells were washed. PC12 cells were treated with A23187, and the culture was continued for the indicated time periods. An aliquot of each culture's supernatants were taken, and the radioactivity was measured (*E*). After 48 h transfection, fresh medium was added with [^3^H] DA. DA uptake was measured after 2 h incubation at 37°C. The results represent the mean ± S.D. of three independent experiments (*F*). *p<0.05 compared with scramble control.

Next, we carried out experiments to determine whether Hsp60 affects N-SMase activity and ceramide production. PC12 cells were transfected with scramble or Hsp60 siRNA and were subsequently analyzed for N-SMase activity, N-SMase2 mRNA levels, and ceramide levels. Hsp60 knockdown effectively increased Mg^2+^-dependent N-SMase activity (∼25%; Figure 9A) and ceramide production (∼20%; Figure 9C). Additionally, we determined the levels of various endogenous ceramides. C16- and C24-ceramide levels were increased significantly by knockdown Hsp60 (Figure 9D). However, treatment with Hsp60 siRNA did not change N-SMase2 mRNA levels (Figure 9B). Hence, Hsp60 knockdown increased N-SMase activity and ceramide levels without induction of N-SMase2 mRNA.

Because of the above results, which showed that Hsp60 siRNA transfection increased N-SMase activity, we next investigated the influence of the increased N-SMase activity on cell death. The viability and cytotoxicity of both scramble- and siRNA-transfected cells were similar (data not shown). In addition, the caspase-3 activity in the Hsp60 siRNA-transfected PC12 cells did not differ significantly from that in the scramble-transfected cells or the negative control.

### Effect of Hsp60 siRNA on dopamine transmission in PC12 cells

Our previous reports suggested that N-SMase may be involved in the dopaminergic system in concert with Ca^2+^ [20], [26]. To further examine this issue, we transiently transfected Hsp60 siRNA into PC12 cells and measured DA release and uptake. As shown in Figure 9E, Hsp60 knockdown had no significant effect on DA release. However, Hsp60 knockdown significantly increased DA uptake (∼50%), whereas N-SMase2 knockdown significantly reduced DA uptake by 40% as compared to the scramble-treated cells. Double knock-down of Hsp60 and NSMase2 recovered DA uptakes lightly from si-NSMase2 treated only, although there is no significant different (Figure 9F). This effect reflects the specific effect of ceramide produced by N-SMase on DA uptake.

### Role of Hsp60 in regulation of N-SMase2 activity

**Figure 10:**
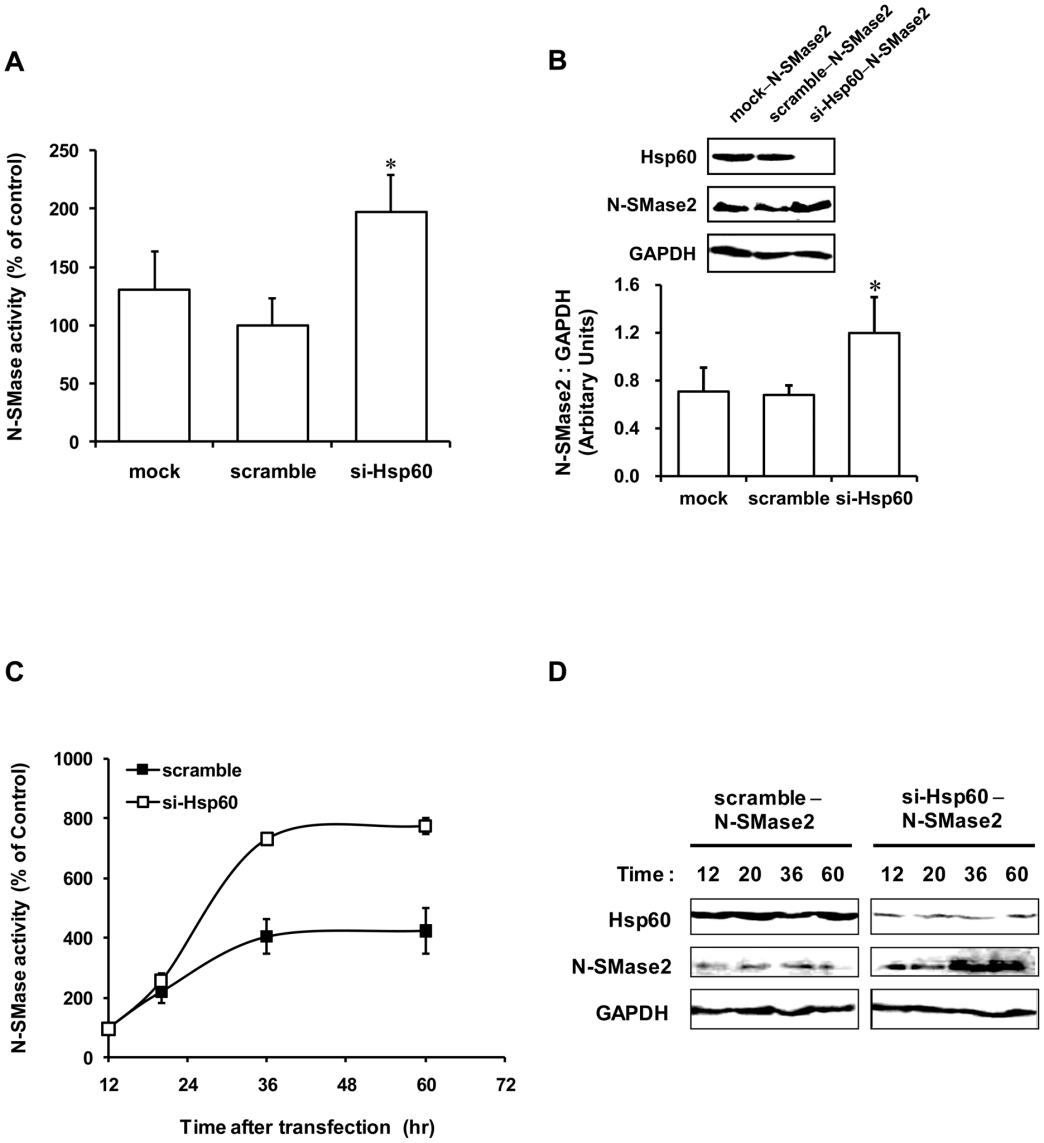
Hsp60 siRNA increases N-SMase activity in N-SMase2–overexpressed HEK293 cells. HEK293 cells were seeded into six-well dishes, and 24 h later, they were transfected with the negative control (mock and scramble) or Hsp60 siRNA (20 nM). After 48 h, the cells were transfected with N-SMase2 for 24 h. Total protein was extracted, and the N-SMase activity was determined (*A*) followed by immunoblotting for Hsp60, N-SMase2, and GAPDH (*B*). The results represent the mean ± S.D. of five independent experiments. *p<0.05 compared with scramble control. After siRNA transfection for 48 h, the cells were transfected with N-SMase2 and incubated for the indicated time periods. Total protein was extracted, and N-SMase activity was determined (*C*) followed by immunoblotting for Hsp60, N-SMase2, and GAPDH (*D*). These results represent the mean ± S.D. from a single experiment performed in triplicate. Similar results were obtained in three independent experiments.

To further define the interaction between Hsp60 and N-SMase2, HEK293 cells were transfected with Hsp60 or scramble siRNA for 48 h before transient transfection with N-SMase2. In all cases, basal levels of N-SMase activity were unaffected by siRNA treatment (data not shown). However, Hsp60 siRNA significantly increased the activity of N-SMase2 (Figure 10A). Concomitantly, Hsp60 siRNA induced an increase in the intensity of the N-SMase2 band on western blotting (Figure 10B). This result suggests that Hsp60 may regulate the stability of N-SMase2 but that it is not involved in the post-translational activation of N-SMase2.

To confirm whether Hsp60 regulated N-SMase2 stability in HEK293 cells, the time course of N-SMase activity induced by N-SMase2 overexpression in the Hsp60 siRNA-transfected cells was measured *in vitro*. The results indicated that Hsp60 siRNA treatment induced a significant increase in N-SMase activity after transfection for 20 h (Figure 10C, 10D and Figure S4). Taken together, these results suggest that Hsp60 down-regulation increases N-SMase2 stability.

## Discussion

In this study, a neuronal form of N-SMase from salt extracts of the membrane fractions of bovine brain, termed N-SMase ε, was purified to near homogeneity. On two-dimensional gel electrophoresis, the purified enzymatic activity was represented as different pI values with identical molecular mass. MALDI-TOF analysis of the tryptic peptides revealed that these four protein species (Figure 1D) were identical, and they were commonly identified as Hsp60. Although Hsp60 had a considerable chromatographic profile, Hsp60 is not a structural gene for novel N-SMase (Figure S1). In other purification procedure, the purified N-SMase preparation had components of apparent molecular weight 57, 71 and 82-kDa. We have found significant variation in different preparation of purified enzymes; in extreme cases, gel electrophoresis revealed only the 60-kDa band.

Many groups have attempted to purify, identify, and characterize a membrane-bound N-SMase from different sources, including rat brain [10], liver [33], hepatoma cells [34], and human brain [35]. Thus far, purification efforts have not been very successful, probably due to the highly hydrophobic nature of the integral membrane proteins. Even in this study, N-SMase ε appeared to be highly hydrophobic, since it was only slightly eluted until distilled water or Triton X-100 was used. This hydrophobic property was further confirmed in the gel filtration and ion-exchange column chromatographies; that is, N-SMase ε activity was eluted from the Superose 12 gel filtration column near void volume, regardless of the presence of Triton X-100 (data not shown). Moreover, the enzyme was insufficiently eluted until Triton X-100 was used as a component of the elution buffer in some of the ion-exchange columns. Our results revealed that purified N-SMase ε appears to migrate as a complex on a gel filtration column, suggesting that the enzyme may form a large complex with other proteins.

According to the results of the purification experiment, the purified enzyme was identified as N-SMase2 based on biochemical properties and western blotting analysis. The activity of this highly purified N-SMase was dependent upon the cations Mg^2+^ and Mn^2+^ and was enhanced by PS, similar to the activity of N-SMase2 (Figure 2). Moreover, there was a strong correlation between the enzyme activity during the purification process and the density of the bands detected by western blotting using anti-N-SMase2 antibody (Figure 3). These findings also strongly support the suggestion that the purified N-SMase ε is N-SMase2. Therefore, we investigated the possibility of an interaction between Hsp60 and N-SMase2 and identified Hsp60 as a component of a multiprotein complex that comprised N-SMase2.

Several lines of evidence support the notion that the interaction between Hsp60 and N-SMase2 is physiologically relevant. First, Hsp60 was co-purified with N-SMase2 through the chromatographic procedure despite the stringent washing conditions employed during the purification processes (Figure 3). Second, the proximity ligation assay for N-SMase2 and Hsp60 showed their colocalization (Figure 8B). Furthermore, Hsp60 was co-immunoprecipitated with N-SMase2 from the purified fraction and extracts of N-SMase2-overexpressed cells (Figure 4 and 6). Third, immunoprecipitates from the anti-Hsp60 antibody had biochemical properties similar to those of N-SMase2 (Figure 5).

The known functions of chaperonins include folding, assembly, and translocation of other proteins [36], [37], [38]. In addition to these housekeeping and cytoprotective roles, signal transduction functions of Hsps have been revealed. Other functions of Hsps have also been shown to include involvement in several processes during synaptic transmission; for example, Hsc70 is essential for uncoating synaptic vesicles at presynaptic terminals [39], and Hsp90 is involved in the synaptic cycling of AMPA receptors [40]. Moreover, constitutively expressed Hsp90, Hsc70, Hsp40, Hsp60, and a range of neurotransmitter receptors are associated with lipid rafts isolated from the rat forebrain and cerebellum [32].

The observations presented herein suggest that N-SMase2 interacts with the chaperone protein Hsp60 in the brain synaptosome and in PC12 cells and that this interaction is important in the maintenance of N-SMase2 protein levels. In our results, while N-SMase2 knockdown effectively decreased N-SMase activity, ceramide levels, and N-SMase2 mRNA levels, Hsp60 knockdown increased Mg^2+^-dependent N-SMase activity and ceramide production in PC12 cells (Figure 9A-D). However, Hsp60 siRNA transfection did not alter N-SMase2 mRNA levels (Figure 9B), also suggesting that the observed increase in N-SMase activity may be due to the reduction in protein degradation rather than to an increase in transcription/translation. This conclusion is further supported by similar results from HEK293 cells. Hsp60 siRNA treatment significantly increased N-SMase activity and the corresponding band in western blotting analysis in N-SMase2-overexpressed HEK293 cells (Figure 10). Therefore, to clarify this aspect of cellular regulation of N-SMase2, although the effect of Hsp60 on the maturation and degradation of N-SMase2 and corresponding levels of protein and activity remains to be studied, one possible role of Hsp60 seems to be involvement in the stability mechanism of N-SMase2 protein.

It has been shown that the brain contains the highest level of N-SMase activity, suggesting that N-SMase may regulate a brain-specific process [17]. N-SMase2 is one of various forms of N-SMase existed abundantly in the brain [15], [18] and has been implicated in apoptosis, inflammation, and cell growth [19]. However, the precise roles of these enzymes in the brain and the mechanisms by which they function remain incompletely understood.

DA is the predominant catecholamine neurotransmitter in the CNS. Dopaminergic neurotransmission in the brain plays a central role in the control of movement, hormone release, and many complex behaviors [20]. Disruptions of DA signaling contribute to various psychiatric and neurological disorders, including drug addiction, schizophrenia, and Parkinson's disease [41], [42]. DA re-uptake through DA transporter (DAT) into presynaptic terminals is thought to be a key step in the termination of neurotransmission [43], [44]. PC12 cells are known to be a useful model of such neuronal systems and include dopamine transporters, but the role of ceramide in DA uptake in the dopaminergic neuron remains unclear. In our previous study, we demonstrated that exposure of PC12 cells to synthetic C_6_-ceramide caused a dose-dependent increase in DA re-uptake. Moreover, N-SMase2 siRNA treatment significantly suppressed DA uptake via its regulation of ceramide production and the resulting ceramide-dependent decrease of intracellular calcium [20]. In the present study, we investigated the effects of Hsp60 on N-SMase2–mediated neuronal regulation, particularly DA uptake. Although N-SMase2 siRNA significantly reduced DA uptake by 40%, Hsp60 knockdown significantly increased DA uptake (∼50%) (Figure 9F) compared with scramble-treated cells. Together, these results reveal an important link between Hsp60 and N-SMase2 in modulation of neuronal activity.

DAT trafficking on the cell surface is critical to DA signaling/homeostasis. This process is known to be regulated by receptor signaling, by direct activation of protein kinase C [45], [46], [47], [48], and by interaction with cytosolic proteins [49], [50], [51]. In addition, recent studies have shown that DAT function can be regulated through a direct protein–protein interaction by intracellular proteins such as α-synuclein, PICK1, synaptogyrin-3 and dopamine D2 receptor [49], [52], [53], [54]. These interactions suggest that the synaptic distribution, targeting, compartmentalization, trafficking, and functional properties of DAT could be regulated via protein–protein interactions. Although our present study suggests that Hsp60 may be involved in regulating DAT function via ceramide production through N-SMase2, the molecular pathway underlying the functional modulation of DAT by Hsp60 has not been identified. Further studies are needed to distinguish between effects mediated by a direct action of N-SMase2 and an indirect effect of N-SMase2-induced intracellular Ca^2+^ levels.

In summary, we identified Hsp60 as a component of a protein complex that is bound with N-SMase ε activity, which was finally defined as N-SMase2, during a procedure purifying N-SMase ε from the membrane fraction of bovine brain tissue. Our results further demonstrated that Hsp60 plays a critical role in dopamine re-uptake through its regulation of N-SMase2 activity and the cellular level of ceramide, possibly by decreasing the stability of the protein level of N-SMase2.

## Supporting Information

Figure S1 **Overexpression of Hsp60 in** ***S. cerevisiae*** **.** Plasmids were transfected into yeast cells using litium acetate method. The expression of N-SMase2 was induced by incubating the cells in synthetic complete-Ura medium containing 2% galactose overnight. Yeast cells were disrupted with glass beads. Glass beads and cell debris were removed by centrifugation at 2,000× g for 10 min, and the supernatant was used for N-SMase activity determinations.
(TIF)

Figure S2 **Immunoprecipitation of Hsp60 and N-SMase 2 from N-SMase2 siRNA-knock downed PC12 cells.** PC12 cells were seeded in 6 well dish and 24 h later were transfected with N-SMase2 siRNA. After 48 h, Immunoprecipitation were carried out using antibodies against Hsp60 or N-SMase2. (*A*) Aliquots of the immunoprecipitated pellets were washed and assayed for N-SMase activity. (*B*) Immunoprecipitated pellets of siRNA-untreated PC12 cell were separated by SDS-PAGE and immunobloted by anti-N-SMase2 and anti-Hsp60 antibody.
(TIF)

Figure S3 **Determination of co-localization of Hsp60 and N-SMase2 using proximity ligation assay in HEK293 cells.** Immunofluorescent confocal microscopy in combination with in situ proximity ligation assay was used to detect and visualize Hsp60/N-SMase2 interaction in HEK293 cells which were transfected with N-SMase2. Irrespective of intensity each red dot represents a single endogenous Hsp60 protein in dose proximity to a single endogenous N-SMase2 protein. DNA counterstained with DAPI (blue).
(TIF)

Figure S4 **Time course of N-SMase activity induced by Hsp60 siRNA treatment in N-SMase2-overexpressed HEK293 cells** HEK293 cells were seeded in 6-well dishes and 24 h later the cells were transfected with N-SMase2. After 48 h, the cells were transfected with scramble control or Hsp60 siRNA (20 nM). Total proteins were extracted, and N-SMase activity (*A*) and immunoblotting (*B*) were analyzed. The results represent the mean ± S.E. of three independent experiments.
(TIF)
