# Supplementary figures and images for: Identification of Heat Shock Protein 60 as a Regulator of Neutral Sphingomyelinase 2 and Its Role in Dopamine Uptake
Source: PLoS One. 2013 Jun 19;8(6):e67216. doi: 10.1371/journal.pone.0067216 (PMC3686747; doi:10.1371/journal.pone.0067216)

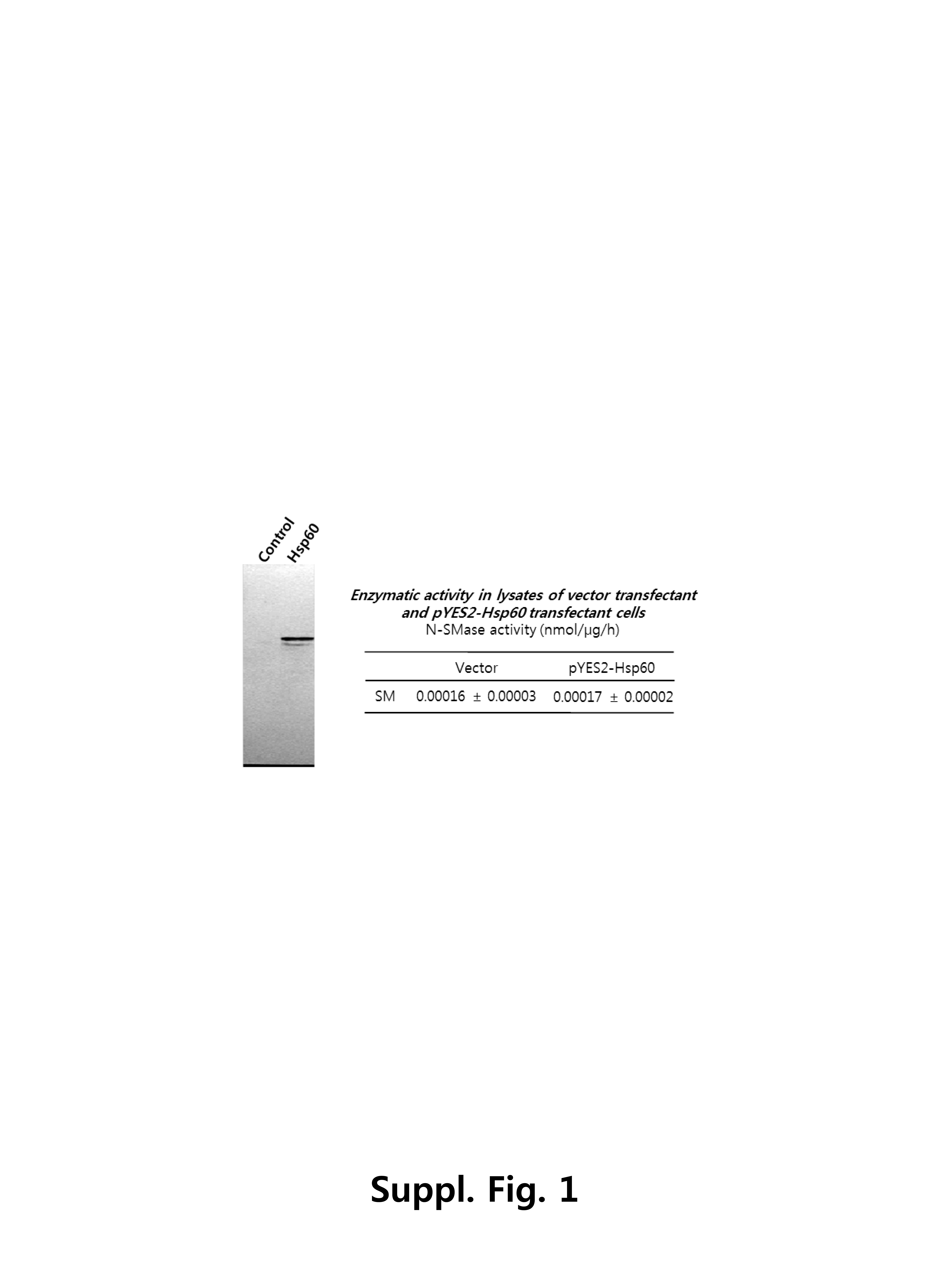

Supplement: Figure S1 — Overexpression of Hsp60 in S. cerevisiae . Plasmids were transfected into yeast cells using litium acetate method. The expression of N-SMase2 was induced by incubating the cells in synthetic complete-Ura medium containing 2% galactose overnight. Yeast cells were disrupted with glass beads. Glass beads and cell debris were removed by centrifugation at 2,000× g for 10 min, and the supernatant was used for N-SMase activity determinations. (TIF) [file pone.0067216.s001.tif]

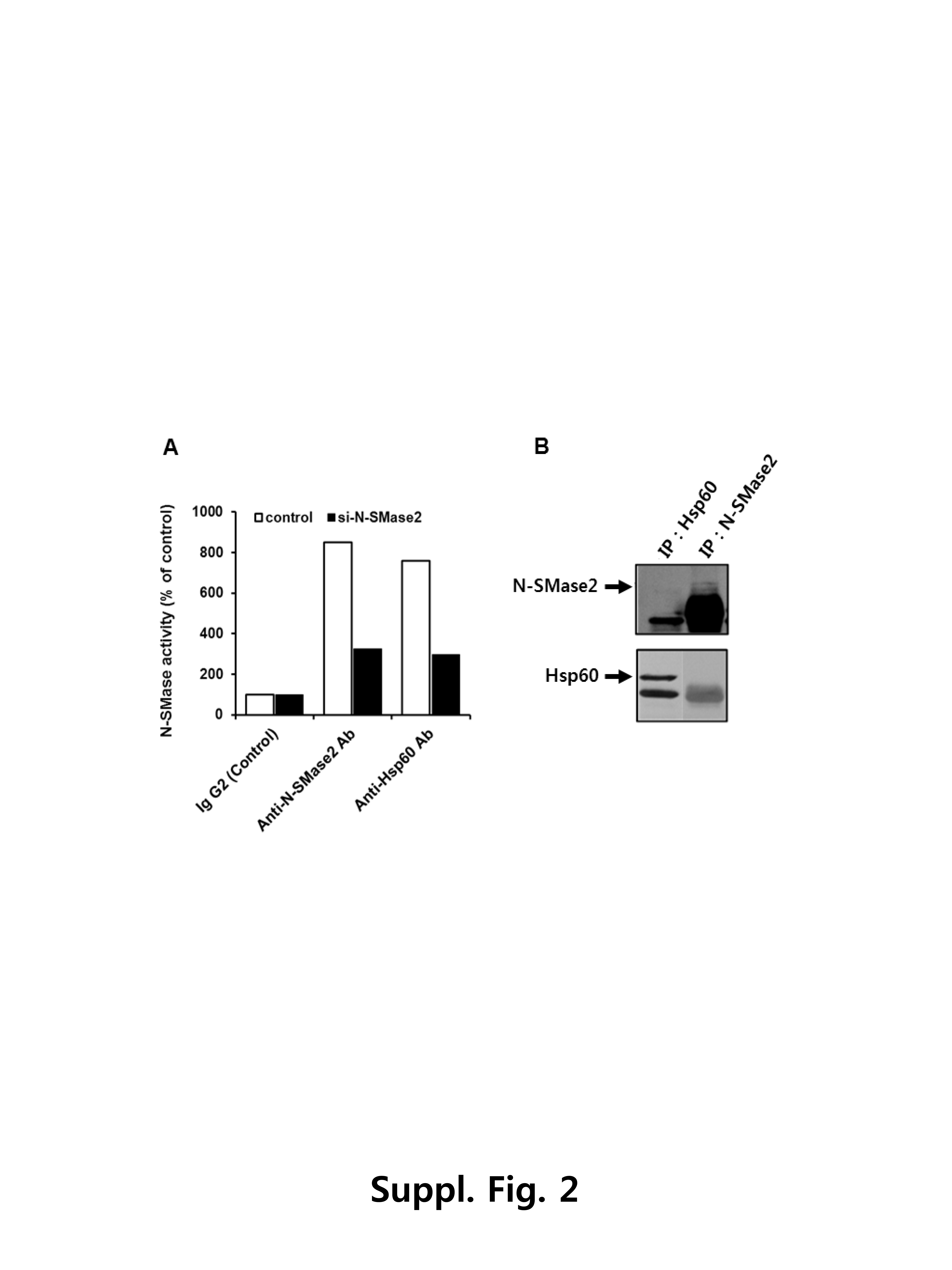

Supplement: Figure S2 — Immunoprecipitation of Hsp60 and N-SMase 2 from N-SMase2 siRNA-knock downed PC12 cells. PC12 cells were seeded in 6 well dish and 24 h later were transfected with N-SMase2 siRNA. After 48 h, Immunoprecipitation were carried out using antibodies against Hsp60 or N-SMase2. (A) Aliquots of the immunoprecipitated pellets were washed and assayed for N-SMase activity. (B) Immunoprecipitated pellets of siRNA-untreated PC12 cell were separated by SDS-PAGE and immunobloted by anti-N-SMase2 and anti-Hsp60 antibody. (TIF) [file pone.0067216.s002.tif]

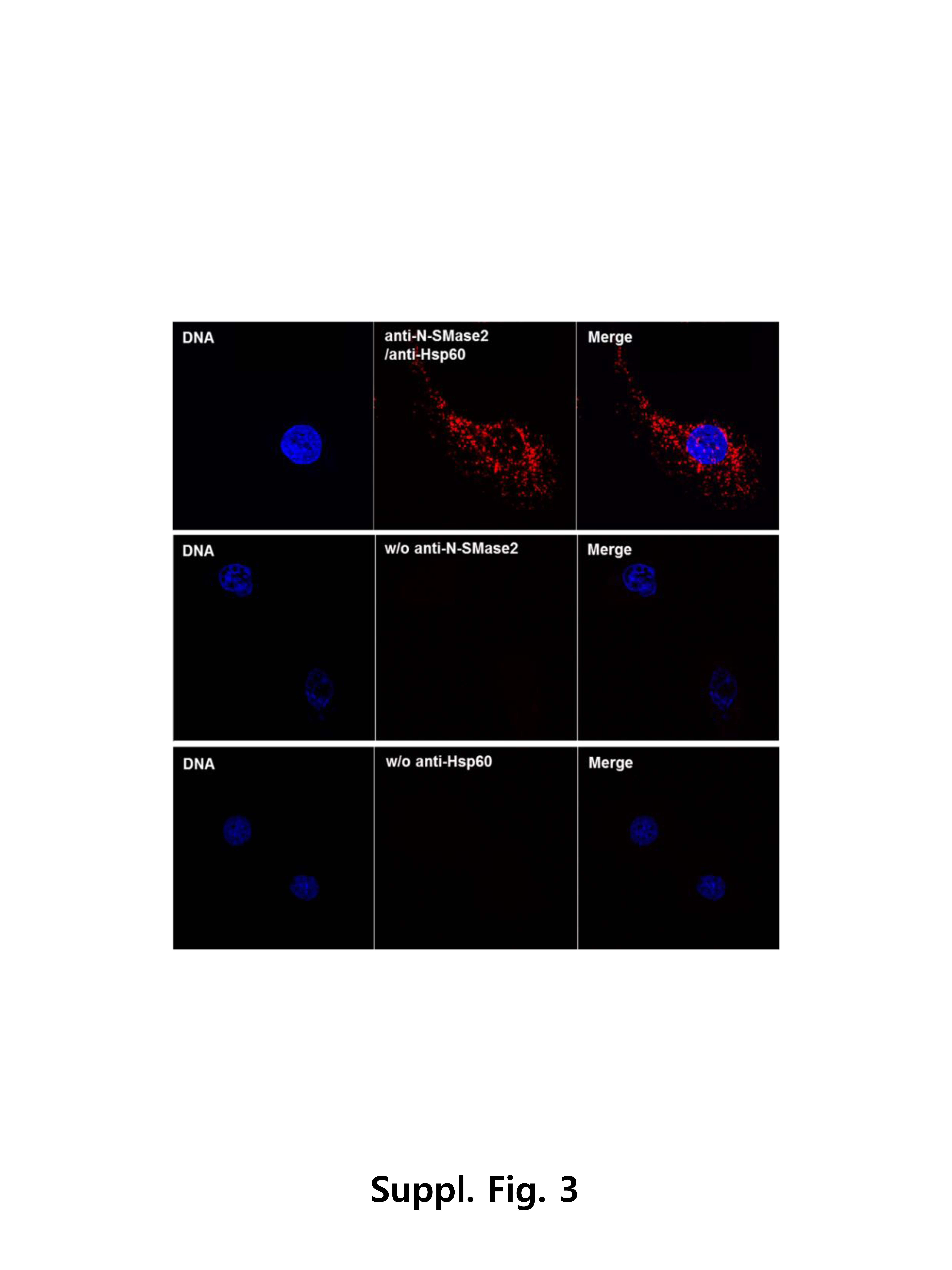

Supplement: Figure S3 — Determination of co-localization of Hsp60 and N-SMase2 using proximity ligation assay in HEK293 cells. Immunofluorescent confocal microscopy in combination with in situ proximity ligation assay was used to detect and visualize Hsp60/N-SMase2 interaction in HEK293 cells which were transfected with N-SMase2. Irrespective of intensity each red dot represents a single endogenous Hsp60 protein in dose proximity to a single endogenous N-SMase2 protein. DNA counterstained with DAPI (blue). (TIF) [file pone.0067216.s003.tif]

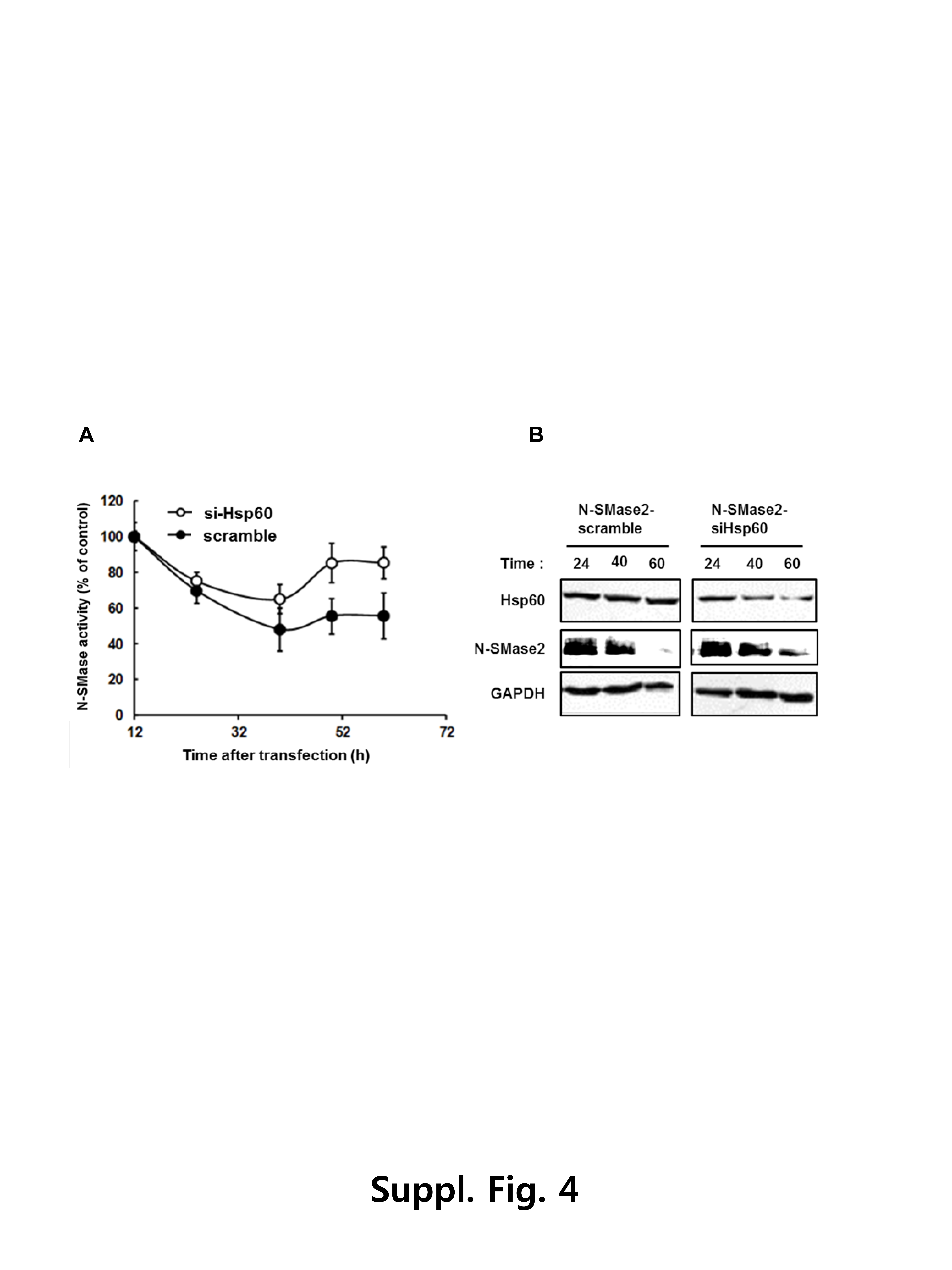

Supplement: Figure S4 — Time course of N-SMase activity induced by Hsp60 siRNA treatment in N-SMase2-overexpressed HEK293 cells HEK293 cells were seeded in 6-well dishes and 24 h later the cells were transfected with N-SMase2. After 48 h, the cells were transfected with scramble control or Hsp60 siRNA (20 nM). Total proteins were extracted, and N-SMase activity (A) and immunoblotting (B) were analyzed. The results represent the mean ± S.E. of three independent experiments. (TIF) [file pone.0067216.s004.tif]
